# Convolutional neural network optimizes the application of diffusion kurtosis imaging in Parkinson’s disease

**DOI:** 10.1186/s40708-021-00139-z

**Published:** 2021-09-28

**Authors:** Junyan Sun, Ruike Chen, Qiqi Tong, Jinghong Ma, Linlin Gao, Jiliang Fang, Dongling Zhang, Piu Chan, Hongjian He, Tao Wu

**Affiliations:** 1 Department of Neurobiology, Neurology and Geriatrics, Xuanwu Hospital of Capital Medical University, National Clinical Research Center for Geriatric Disease, Beijing, 100053 China; 2 Center for Brain Imaging Science and Technology, College of Biomedical Engineering and Instrumental Science, Zhejiang University, Hangzhou, 310027 Zhejiang China; 3 Research Center for Healthcare Data Science, Zhejiang Lab, Hangzhou, Zhejiang China; 4 Department of Neurology, Xuanwu Hospital of Capital Medical University, Beijing, China; 5 Department of Radiology, Guang’anmen Hospital, China Academy of Chinese Medical Sciences, Beijing, China; 6 Clinical Center for Parkinson’s Disease, Capital Medical University, Beijing, China; 7 Key Laboratory for Neurodegenerative Disease of the Ministry of Education, Beijing Key Laboratory for Parkinson’s Disease, Parkinson Disease Center of Beijing Institute for Brain Disorders, Beijing, China; 8 National Clinical Research Center for Geriatric Disorders, Beijing, China; 9 Key Laboratory for Biomedical Engineering of Ministry of Education, Zhejiang University, Hangzhou, 310027 Zhejiang China

**Keywords:** Parkinson’s disease, Diffusion kurtosis imaging, Convolutional neural network, Mean kurtosis, Kurtosis fractional anisotropy, Mean diffusivity

## Abstract

**Objectives:**

The literature regarding the use of diffusion-tensor imaging-derived metrics in the evaluation of Parkinson’s disease (PD) is controversial. This study attempted to assess the feasibility of a deep-learning-based method for detecting alterations in diffusion kurtosis measurements associated with PD.

**Methods:**

A total of 68 patients with PD and 77 healthy controls were scanned using scanner-A (3 T Skyra) (DATASET-1). Meanwhile, an additional five healthy volunteers were scanned with both scanner-A and an additional scanner-B (3 T Prisma) (DATASET-2). Diffusion kurtosis imaging (DKI) of DATASET-2 had an extra *b* shell compared to DATASET-1. In addition, a 3D-convolutional neural network (CNN) was trained from DATASET-2 to harmonize the quality of scalar measures of scanner-A to a similar level as scanner-B. Whole-brain unpaired *t *test and Tract-Based Spatial Statistics (TBSS) were performed to validate the differences between the PD and control groups using the model-fitting method and CNN-based method, respectively. We further clarified the correlation between clinical assessments and DKI results.

**Results:**

An increase in mean diffusivity (MD) was found in the left substantia nigra (SN) in the PD group. In the right SN, fractional anisotropy (FA) and mean kurtosis (MK) values were negatively correlated with Hoehn and Yahr (H&Y) scales. In the putamen (Put), FA values were positively correlated with the H&Y scales. It is worth noting that these findings were only observed with the deep learning method. There was neither a group difference nor a correlation with clinical assessments in the SN or striatum exceeding the significance level using the conventional model-fitting method.

**Conclusions:**

The CNN-based method improves the robustness of DKI and can help to explore PD-associated imaging features.

**Supplementary Information:**

The online version contains supplementary material available at 10.1186/s40708-021-00139-z.

## Introduction

Parkinson's disease (PD) is a common neurodegenerative disease characterized by bradykinesia, resting tremor, rigidity, postural balance disturbance, and non-motor manifestations [1]. Beyond the deficiency of dopaminergic neurons and aggregation of Lewy bodies in the basal ganglia, pathological changes in PD are associated with axonal lesions and synaptic dysfunction, which contribute to the impairment of white matter integrity [2]. Given the limitation of discerning the intrinsic details and pathological heterogeneity in brain tissues, it is challenging to identify PD-associated microstructural changes using conventional magnetic resonance imaging (MRI). Diffusion-weighted MRI techniques, such as diffusion-tensor imaging (DTI), can non-invasively probe the microstructural properties via the diffusion of water molecules in vivo [3–7]. It has been reported that DTI-derived metrics, such as fractional anisotropy (FA) and mean diffusivity (MD), showed significant differences in the substantia nigra (SN) and some white matter areas between PD patients and controls [8]. Furthermore, diffusion kurtosis imaging (DKI), which is based on DTI and considers the non-Gaussian diffusion of water molecules, was reported as a more sensitive technique to evaluate the pathological characteristics of PD patients [3, 8].

However, previous studies have yielded inconsistent or controversial findings. For example, while some studies reported decreased FA, increased MD, and/or increased mean kurtosis (MK) values in the SN [6, 9–11], other studies [6, 12] observed increased FA values in the SN in PD patients. It has been reported that the FA value of the SN was higher in PD patients than in healthy controls (HCs) [11]. Additionally, Kamagata et al. found decreased MK and FA values of white matter, such as the inferior fronto-occipital fasciculus (IFOF), anterior corona radiata (ACR), and superior longitudinal fasciculi (SLF) [7], while Wen et al. showed increased FA values of the IFOF and bilateral SLF in tremor-dominant PD patients [13].

It is speculated that the heterogeneity of PD patients being recruited and various acquisition protocols of diffusion MRI scanning may have contributed to these controversial findings [4, 5, 8, 14]. DKI can sensitively reflect microstructural complexity, particularly in isotropic tissues such as the gray matter [15]. However, because the gray matter microstructure lacks evident directionality, diffusion-weighted imaging (DWI) signals can be easily affected by noise and limited spatial resolution [16], thus leading to inaccurate findings of alterations in DKI scalar measures. Another limitation to the wide application of DKI is that high *b*-value diffusion signals, which are required for the accurate calculation of DKI scalar measures, are often difficult to obtain in clinical settings.

Recently, deep learning, an important branch of machine learning, has shown significant potential for improving the performance of neuroimaging findings [17–21]. As one of the representative algorithms of deep learning, convolutional neural network (CNN) adopts convolution and down-sampling to certain layers with less computation; adjusts the network weights through the back-propagation and stochastic gradient descent algorithm; recognizes the features or patterns of the raw imaging inputs automatically; and then achieves the classification, identification, and prediction of inputs [21–23].

Li et al. [22] recently proposed a three-dimensional hierarchical CNN (3D H-CNN) to improve the estimation of DKI scalar measures from limited diffusion-weighted (DW) images. Three-dimensional convolution kernels were introduced to automatically extract and learn the features of the DW-images. Only part scalar measures were of clinical interest instead of the full tensors, and the 3D H-CNN (hereafter called CNN) method makes it possible to complete fast and optimized DKI acquisition within 1 min. This method also considers cross-voxel information, which was confirmed to provide enhanced efficiency for estimating DKI scalar measures and improved robustness against noise.

Therefore, in the current study, we aimed to use this CNN-based method to improve the estimation of DKI scalar measures and to determine whether the improved measures can help to delineate PD-associated imaging features.

## Materials and methods

### Participants

Sixty-eight patients with PD who met the Movement Disorder Society clinical diagnostic criteria for PD were recruited from the Movement Disorders Clinic of the Xuanwu Hospital of Capital Medical University. We recruited 77 HCs who met the following criteria: (1) aged ≥ 40 years; (2) no history of neurological or psychiatric diseases; (3) no family history of neurodegenerative disorders, and (4) no apparent cerebral lesions on structural MRI. The Movement Disorder Society Unified Parkinson’s Disease Rating Scale, part III (MDS- UPDRS III) and Hoehn and Yahr (H&Y) scale were performed in all PD patients while they were in the off-state. Their demographic details are summarized in Table 1. In addition, seven healthy volunteers (M/F = 1/6, age = 26.4 ± 1.6 years) were recruited and their DKI data were collected using two different MRI scanners. This experiment was guided by and adhered to the Declaration of Helsinki and was approved by the Institutional Review Board of Xuanwu Hospital. All included participants have signed informed consent before the experiment.

**Table 1:** Demographic and clinical assessments of subjects

	PD (*n* = 68)	HC (*n* = 77)	*p* value
Age in years, mean (SD)	58.94 (8.969)	59.58 (8.537)	0.659
Gender(M/F)^§^	36/32	30/47	0.092
Education in years, mean(SD)	11.81 (3.316)	11.58 (4.143)	0.074
H&Y score, median(range)	2 (1–3)	0	NA
UPDRS III, mean (SD)	26.5 (12.261)	NA	NA
Duration in years, Median (range)	4 (0.5–20)	NA	NA

*M* male, *F* female, *SD* standard variation, *NA* not applicable

^§^Pearson’s Chi-square test

### MRI

#### DATASET-1

For all PD patients and HCs, MRI data were acquired using a 3-T scanner-A (MAGNETOM Skyra, Siemens, Germany) equipped with a 20-channel receiver head and neck joint coil (opening 16 channels). DW-images were obtained in axial orientation using single-shot spin-echo echo-planar imaging sequences (SE-EPI). Diffusion weightings of *b* = 1000 and 2000s/mm^2^ were applied along 30 noncollinear sensitive gradient directions. One *b* = 0 image was acquired, resulting in a total of 61 DW-images. The other imaging parameters were as follows: repetition time (TR) = 5000 ms, echo time (TE) = 105 ms, resolution = 2 × 2 × 2 mm, field of view (FOV) = 220 × 220 mm, number of slices = 68.

#### DATASET-2

Seven healthy volunteers were scanned using two different MRI scanners. We randomly included five volunteers in the CNN training dataset and assigned the remaining two volunteers to the test dataset. Scan 1 also used scanner-A and the same scanning process as used for DATASET-1. For implementation of the CNN, DW-images were also acquired in Scan 2 using a 3-T scanner-B (MAGNETOM Prisma, Siemens Germany) equipped with a 64-channel RF coil. DW-images were obtained using a simultaneous multi-slice diffusion echo-planar imaging sequence (SMS-EPI). Diffusion weightings of *b* = 1000, 2000, and 3000 s/mm^2^ were applied in 30 gradient directions. Six *b* = 0 images and one *b* = 0 image with an opposite phase encoding direction were acquired, resulting in a total of 96 DW-images. The *b* = 0 image with the reversed phase encoding direction was used to correct the field inhomogeneity-induced distortion. The other imaging parameters were as follows: TR = 3000 ms, TE = 75 ms, resolution = 2 mm × 2 mm × 2 mm, FOV = 220 mm × 220 mm, number of slices = 68.

### Image processing

#### Preprocessing

The preprocessing pipeline for both datasets was mainly based on FSL (FMRIB Software Library, University of Oxford, UK) [24]. *Rician* noise was removed using *dwidenoise* included in MRtrix 3 [25], followed by Gibbs-ring removal. Bulk head movement was corrected using FSL, which linearly aligned each diffusion-weighted image to the first *b* = 0 image. For DATASET-2 and Scan 2, distortion correction was also performed using the *topup* and *eddy* tools [26]. The susceptibility-induced off-resonance field map was first estimated by *topup* using a pair of non-DW (*b* = 0) images acquired with reversed phase encoding directions anterior–posterior and posterior–anterior (AP and PA). It was then fed into *eddy* to correct for eddy current and motion-induced distortion.

#### Model-fitting method

**Fig. 1:**
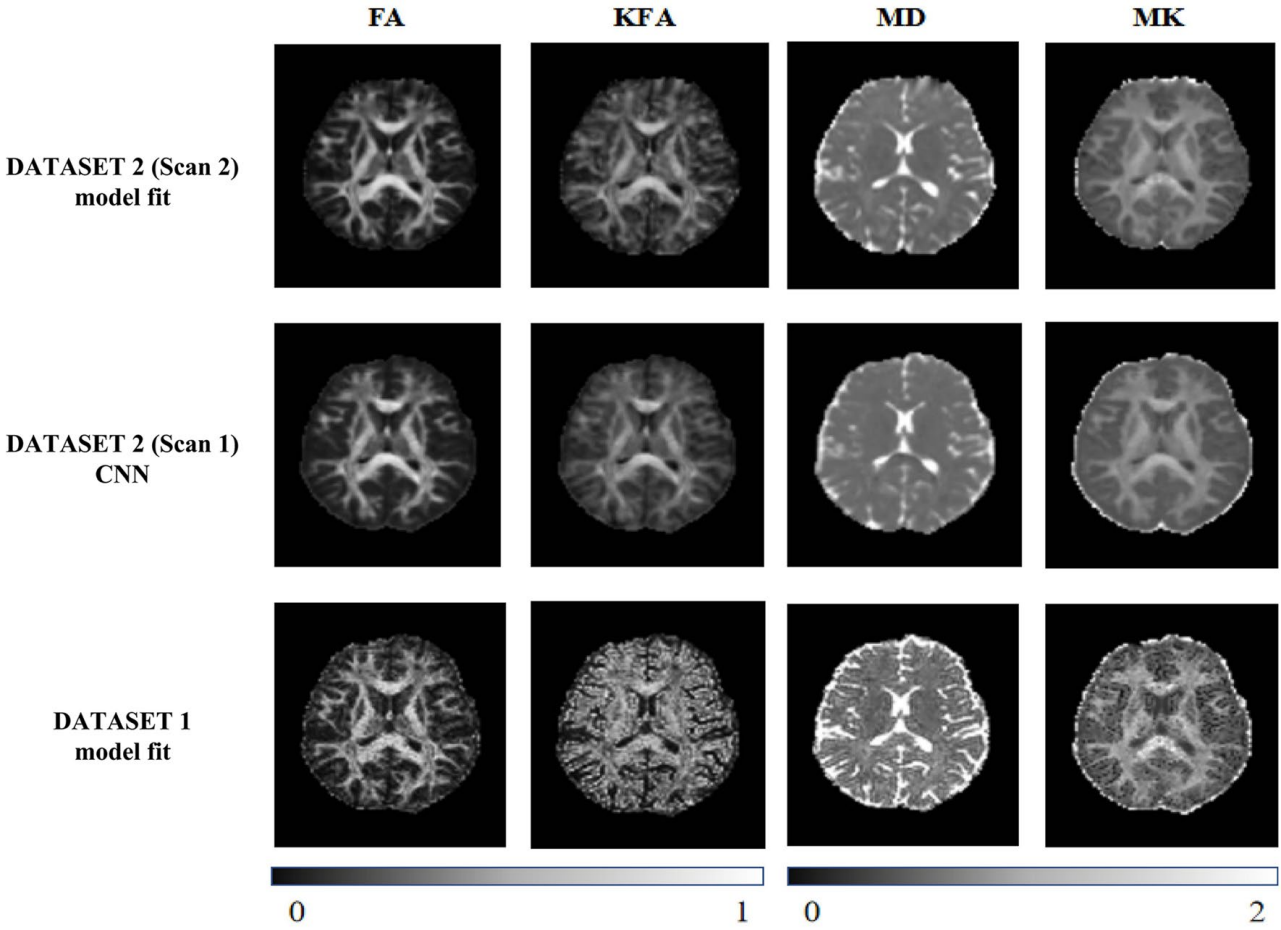
Maps of diffusion kurtosis imaging scalar measures using different methods. Note. High-quality diffusion kurtosis imaging (DKI) scalar maps with the model-fitting method in the first line, moderate-quality DKI scalar maps with the model-fitting method in last line, and moderate quality of DKI scalar maps with the convolutional neural network (CNN) method in the middle line indicate that the CNN-based method can optimize the scalar maps of moderate diffusion-weighted images

For DATASET-1 and Scan 2 of DATASET-2, the model-fitting method was conducted using DESIGNER (diffusion parameter EStImation with Gibbs and NoisE Removal, New York University, US), a post-processing pipeline capable of identifying and correcting various specific artifacts and confounding factors for improved accuracy, precision, and robustness compared to conventional linear least square method fitting (Fig. 1) [27].

#### CNN-based method

A CNN-based method was adopted to improve the estimation of DKI scalar measures from limited-quality DATASET-1 DW-images. The adopted CNN-based method included one input layer, several hidden layers, and two output layers. A dropout layer was inserted before each output layer to prevent overfitting. A 3 × 3 × 3 convolution kernel was introduced in the first hidden layer to extract features from the preprocessed DW-images. The network was constructed using a hierarchical structure. The resulting DKI scalar measures were output through two different layers. The shallow output layer was connected to the penultimate hidden layer and was responsible for scalar measures (FA and MD). Kurtosis-related measures (MK and kurtosis FA) values were output through a deeper layer connected to the final hidden layer [22].

**Fig. 2:**
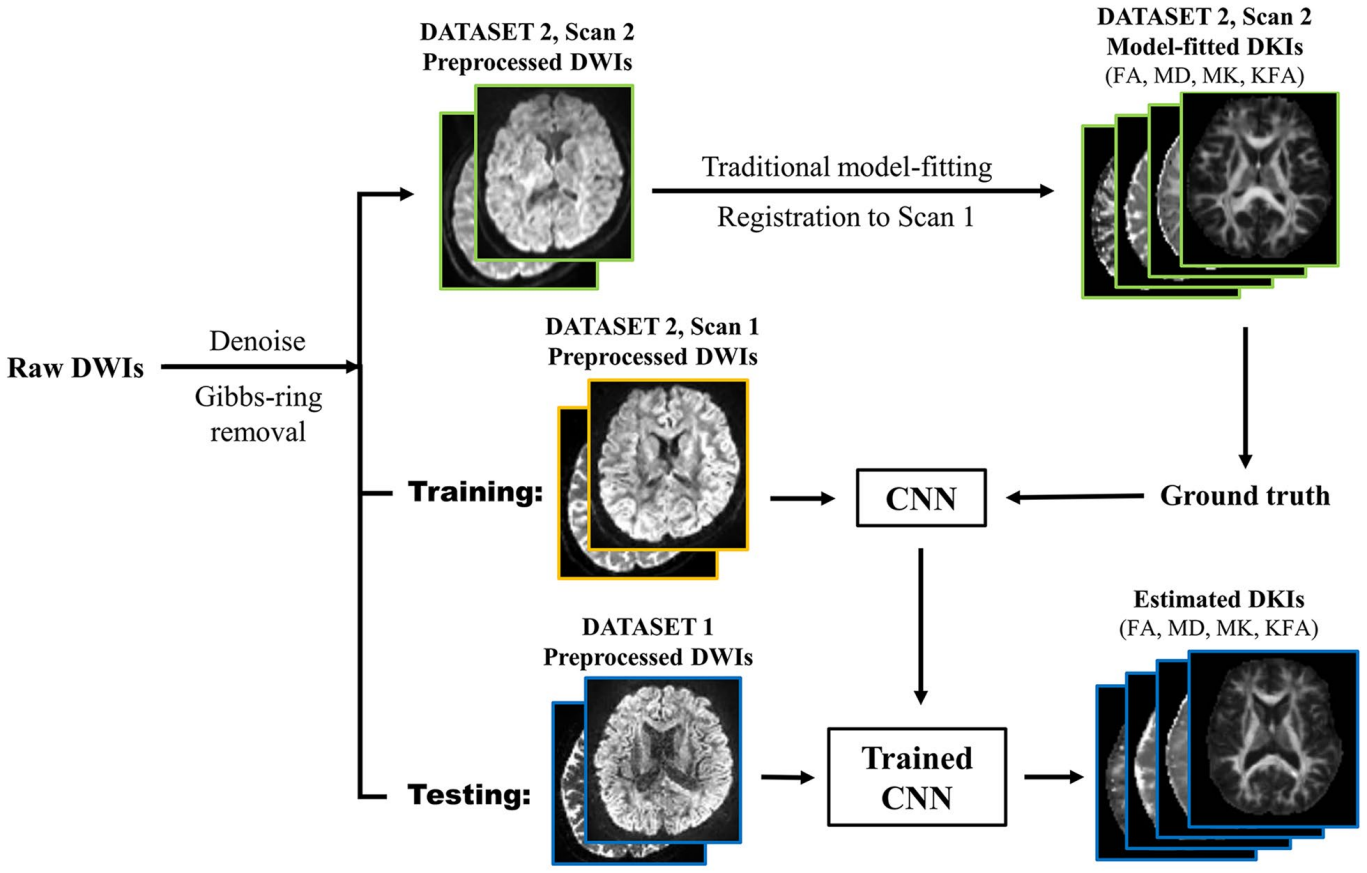
The training and testing pipeline of the convolutional neural network method. Note. CNN, convolutional neural network; DWI, diffusion-weighted imaging; DKI, diffusion kurtosis imaging; FA, fractional anisotropy; MD, mean diffusivity; MK, mean kurtosis; KFA, kurtosis fractional anisotropy

The pipeline of the CNN is shown in Fig. 2. Preprocessed DW-images of DATASET-2, Scan 1 were the training inputs of the CNN and the corresponding model-fitted MK, KFA, FA, and MD metrics for each healthy volunteer in DATASET-2, Scan 2 were defined as the ground truth. Scan 2 was registered to Scan 1 using FNIRT and FSL [28] for every volunteer before training. Specifically, we first removed non-brain tissue from images using BET2 and FSL. Nonlinear registration was then performed using FNIRT and FSL between the *b* = 0 images of Scans 1 and 2 for every volunteer. This resulted in registered images and corresponding nonlinear transformations. We then applied these transformations to the model-fitted DKI of Scan 2 to register them in the same space as Scan 1 images. We checked the registration results visually based on the alignment between the boundaries of the brain structures. We used the DKI data of five healthy volunteers to train the network. The training process was performed for 100 epochs.

We tested the trained CNN on two volunteers (not from the training dataset) and calculated the root-mean-squared error (RMSE) between the CNN-estimated or model-fitted DKI measures and the reference standard:

1 $${\text{RMSE}} = \sqrt {\frac{{\sum\nolimits_{i = 1}^{N} {\left( {\hat{s}_{i} - s_{i} } \right)^{2} } }}{N}} ,$$

where $N$ is the number of voxels in the brain images, ${\widehat{s}}_{i}$ is the CNN-estimated or model-fitted DKI scalar measure in the ${\mathrm{i}}^{\mathrm{th}}$ voxel, and ${s}_{i}$ is the ground truth DKI scalar measure in this voxel. Peak signal-to-noise ratios (PSNRs) were calculated as follows:

2 $${\text{PSNR}} = 10 \times \log_{10} \left( {\frac{{s_{MAX}^{2} }}{MSE}} \right),$$

where ${\mathrm{s}}_{\mathrm{MAX}}$ is the maximum signal value in a DKI scalar image and MSE is the mean-squared error, defined as:

3 $${\text{MSE}} = \frac{{\mathop \sum \nolimits_{i = 1}^{N} \left( {\hat{s}_{i} - s_{i} } \right)^{2} }}{N},$$

**Fig. 3:**
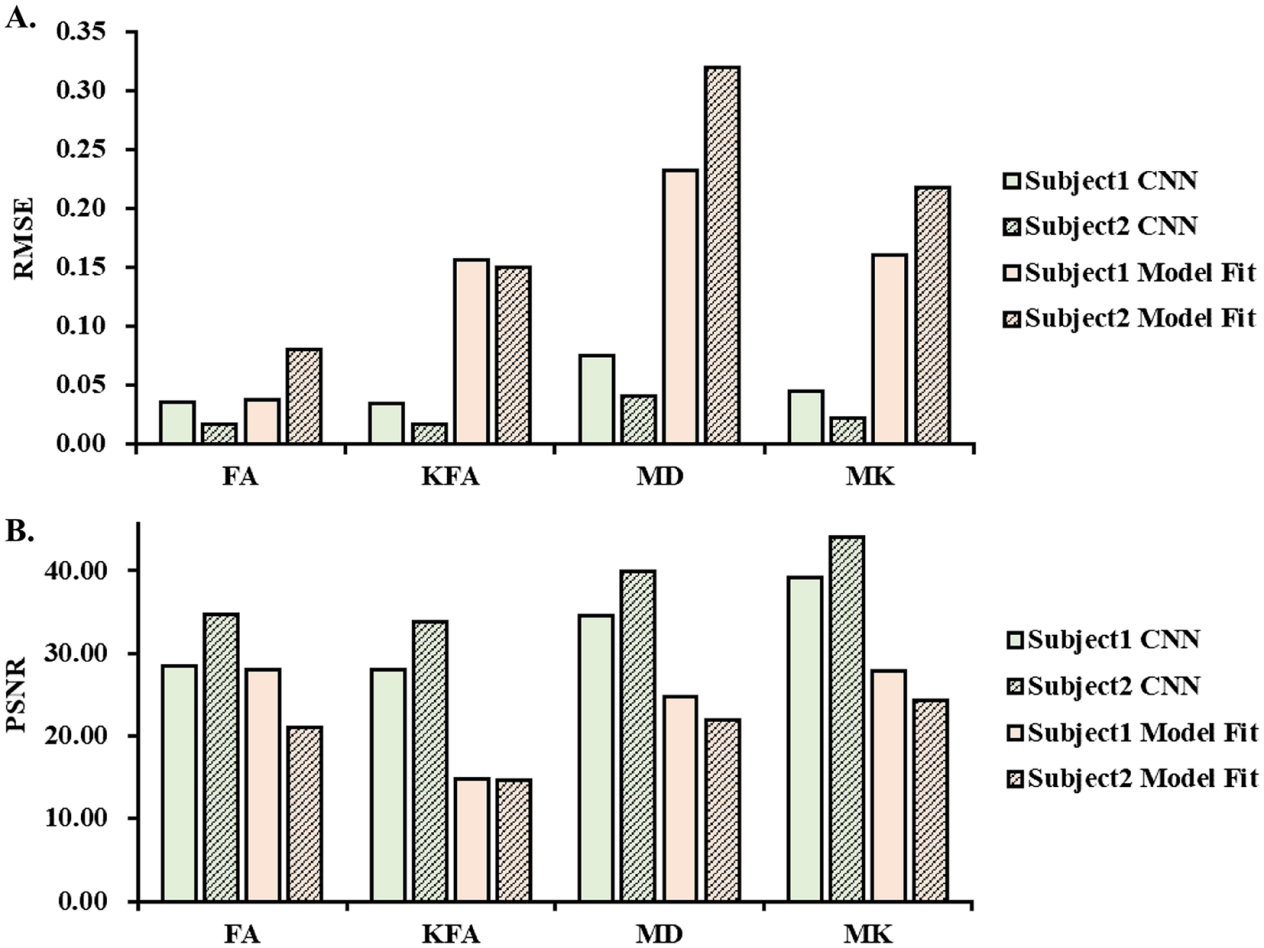
The testing about the accuracy of CNN-estimated measures. Note. PSNR = peak signal-to-noise ratios; RMSE = root-mean-squared error

As shown in Fig. 3, the RMSE results of both testing subjects derived from the CNN-based method were lower than those of the conventional model-fitting method. The DKI scalar images estimated by the trained CNN showed a higher PSNR than the model-fitting method. With the same amount of diffusion-weighted signals acquired, the trained CNN was able to provide DKI estimations with higher qualities and closer to the ground truth.

We performed the same calculations on conventional model-fitted DKI results and compared them with the results of the CNN-based method. Other tests and validations on the robustness of the adopted 3D CNN structure have been described in a previous study [22].

Finally, we applied the trained CNN to subjects in both the HC and PD groups to estimate DKI measures, including FA, MD, KFA, and MK. The preprocessed DW-images from DATASET-1 as described in “Preprocessing” section were used as inputs.

### Statistical analysis

The one-sample Shapiro–Wilk test was used to confirm the normality of each group’s data. The Student’s *t test* and Pearson Chi-square test were used to analyze age and sex variables, respectively.

#### Whole-brain unpaired *t* test

For the DW-images of PD patients and HCs, two datasets of DKI scalar measures were derived using model-fitting and CNN-based methods, respectively. FA maps were first registered to the Montreal Neurological Institute 152 (MNI152) standard space with a resolution of 1 mm isotropic using a combination of linear and nonlinear transforms. The resulting transformation was then applied to all other DKI maps for co-registration. For DKI scalar measures of both methods, whole-brain unpaired t-tests were performed to evaluate the ability of DKI to reveal differences between PD patients and HCs.

#### Tract-based spatial statistics

Furthermore, we performed Tract-Based Spatial Statistics (TBSS) analysis using the model-fitting and CNN-based method, respectively. TBSS extracted the mean FA maps to generate a white matter skeleton, realized by a tool for nonparametric permutation inference implemented in FSL [29].

The threshold-free cluster enhancement [30] based test was included in both the whole-brain unpaired t-test and TBSS analysis to improve robustness compared with conventional voxel-based tests. The number of permutation tests was set to 500 for both TBSS and t-test analyses. All significance thresholds were set at *p* < 0.05 and by family-wise error (FWE)-corrected.

To determine the specific brain regions to which the clusters with significance belonged, we utilized the FSL tool ATLASQUERY. This tool automatically matches the clusters to structural areas in user-specified atlas spaces and outputs the labels of the brain regions. In this study, we referred to parcellations from the Harvard–Oxford Cortical and Subcortical Structural Atlases [31] and JHU DTI-based white matter Atlases [32].

Notably, we determined whether there were intra-group differences in HCs by applying the whole-brain unpaired t-test and TBSS with model-fitted and CNN-based methods, respectively, before comparing differences between the groups.

#### Correlation analysis

To determine the clinical significance of DKI scalar measures using the CNN-based method more clearly, DKI scalar measures (MK, KFA, FA, and MD) determined using the model-fitting or CNN-based method showing significant between-group differences in the basal ganglia (SN, putamen, and caudate) were extracted and correlated with clinical assessments. Pearson’s correlation analysis was used for normally distributed data, and Spearman’s correlation analysis was used for non-normally distributed data. Correlations with significance were defined as *p* < 0.016 (Bonferroni-corrected). Statistical analyses were computed using IBM SPSS Statistics (version 25; IBM Corp., Armonk, NY, USA) and GraphPad Prism 8.0.1.

## Results

### CNN evaluations

As shown in Fig. 1, DKI scalar maps estimated by the CNN for DATASETS-1 had higher signal-to-noise ratios than those obtained by the model-fitting method. Higher-order DKI scalar measures such as KFA showed a clearer contrast between gray and white matter in the CNN-based results. For DATASET-2, the CNN-based results displayed almost the same quality as the reference standard (ground truth). The RMSE and PSNR results are listed in Fig. 3. The RMSE results of both testing subjects derived from the CNN-based method were lower than those from the conventional model-fitting method. The DKI scalar images estimated by the trained CNN showed a higher PSNR than the model-fitting method. With the same amount of diffusion-weighted signals acquired, the trained CNN was able to provide DKI estimations with higher qualities and closer to the ground truth.

### Demographic features

Age (*p* = 0.659, Student’s *t-test*), sex distribution (*p* = 0.092, Pearson Chi-square test), and education (*p* = 0.074, Student’s *t-test*) did not differ between the PD and HC groups. The demographic details are summarized in Table 1.

### Validation of intra-group differences between the PD and HC groups

Before comparing the PD patients and healthy controls, we separated all HCs and PD patients into two age-matched groups, respectively, and then conducted within-group comparisons, and found no significant difference with either the CNN-based or model-fitting method. It is preliminarily ruled out the possibility of false positives in the CNN-based method.

### Whole-brain unpaired t-test analysis

#### Model-fitting method

FA values in the bilateral putamen (Put) and globus pallidus (GP), left caudate (Cau) and accumbens (Acc), bilateral superior corona radiata (SCR) and anterior thalamic radiation (ATR), et al. were higher in PD patients than in HCs.

MD values in bilateral Put, GP, Cau, thalamus (Thal), bilateral cerebral cortex and white matter, bilateral posterior thalamic radiation (PTR) and inferior longitudinal fasciculus (ILF) and genu of corpus callosum (GCC), et al. were significantly increased in PD patients compared to HCs.

There was no significant difference in the MK and KFA values between the groups (*p* < 0.05, FWE-corrected).

**Fig. 4:**
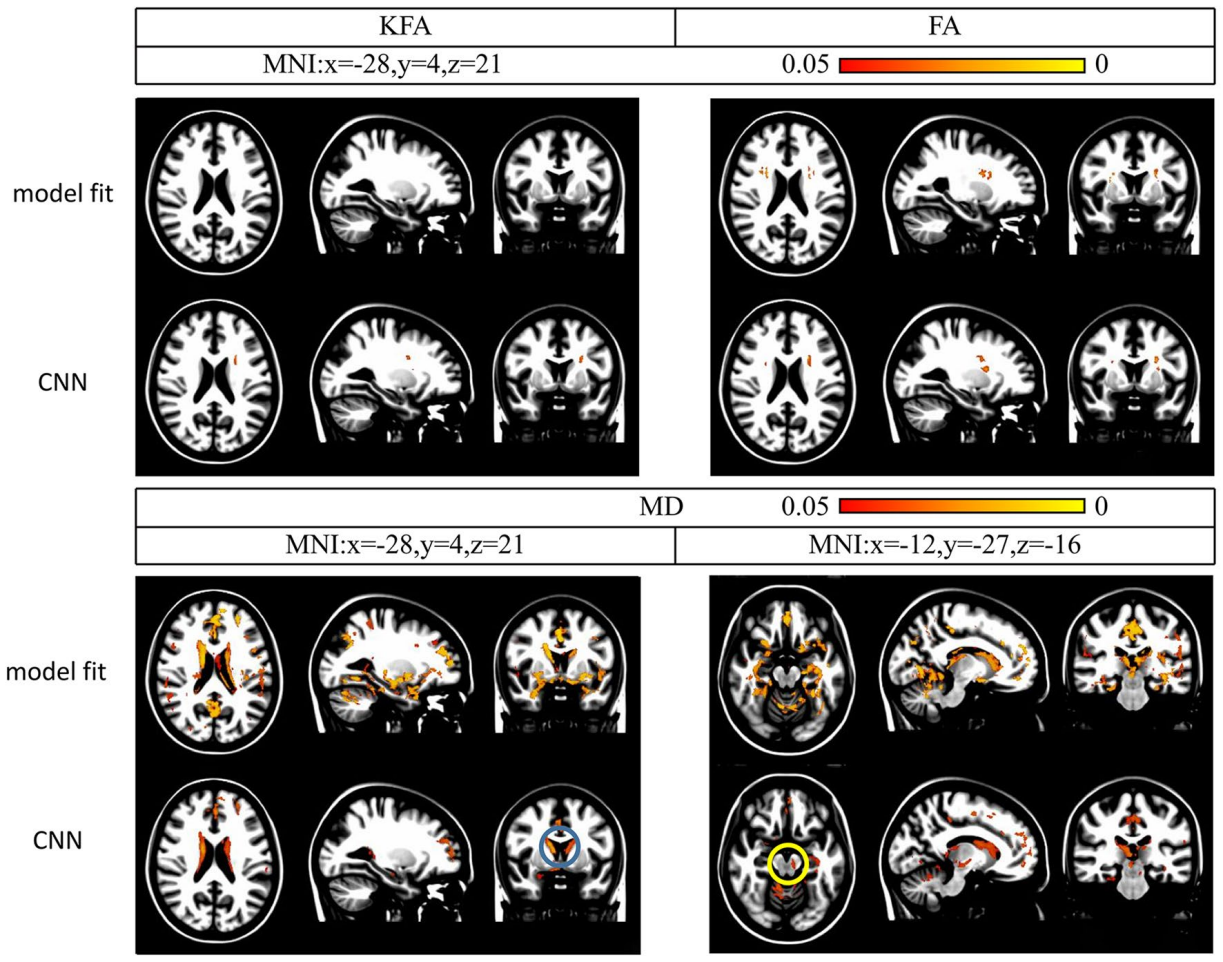
Whole-brain unpaired t-test analysis of diffusion kurtosis imaging measures using the CNN-based and model-fitting methods. Note. Increased mean diffusivity (MD) values in the bilateral caudate (Cau) (blue circle) and right substantia nigra (SN) (yellow circle) with the CNN-based method between the healthy control (HC) and Parkinson’s disease (PD) groups (*p* < 0.05, family-wise error-corrected). KFA, kurtosis fractional anisotropy; FA, fractional anisotropy; MNI, Montreal Neurological Institute

Please check Fig. 4 and Additional file 1: Table S1 for more details.

#### CNN-based method

FA values were increased in left Put, bilateral cerebral cortex and white matter, bilateral SCR and left ACR, et al., while KFA values were increased only in the left ACR and cerebral cortex and white matter in PD patients compared to HCs.

MD values in the left substantia nigra (SN) and left hippocampus and bilateral Cau and Thal, bilateral cerebral cortex and white matter, GCC and bilateral ATR, et al., were clearly increased in PD patients compared to HCs.

There was no significant difference in the MK values between the groups (*p* < 0.05, FWE-corrected).

Please check Fig. 4 and Additional file 1: Table S1 for more details.

### TBSS analysis

#### Model-fitting method

MK values were higher in the left ATR, left IFOF, left ILF, and left uncinate fasciculus (UNC), et al. in PD patients than in HCs.

FA values were increased in the left ATR, left IFOF, left corticospinal tract and left SLF, et al. in PD patients compared to HCs.

Compared to HCs, PD patients showed increased MD values in the bilateral IFOF, left ILF and bilateral UNC, et al.

There was no significant difference in the KFA values between the groups (*p* < 0.05, FWE-corrected).

**Fig. 5:**
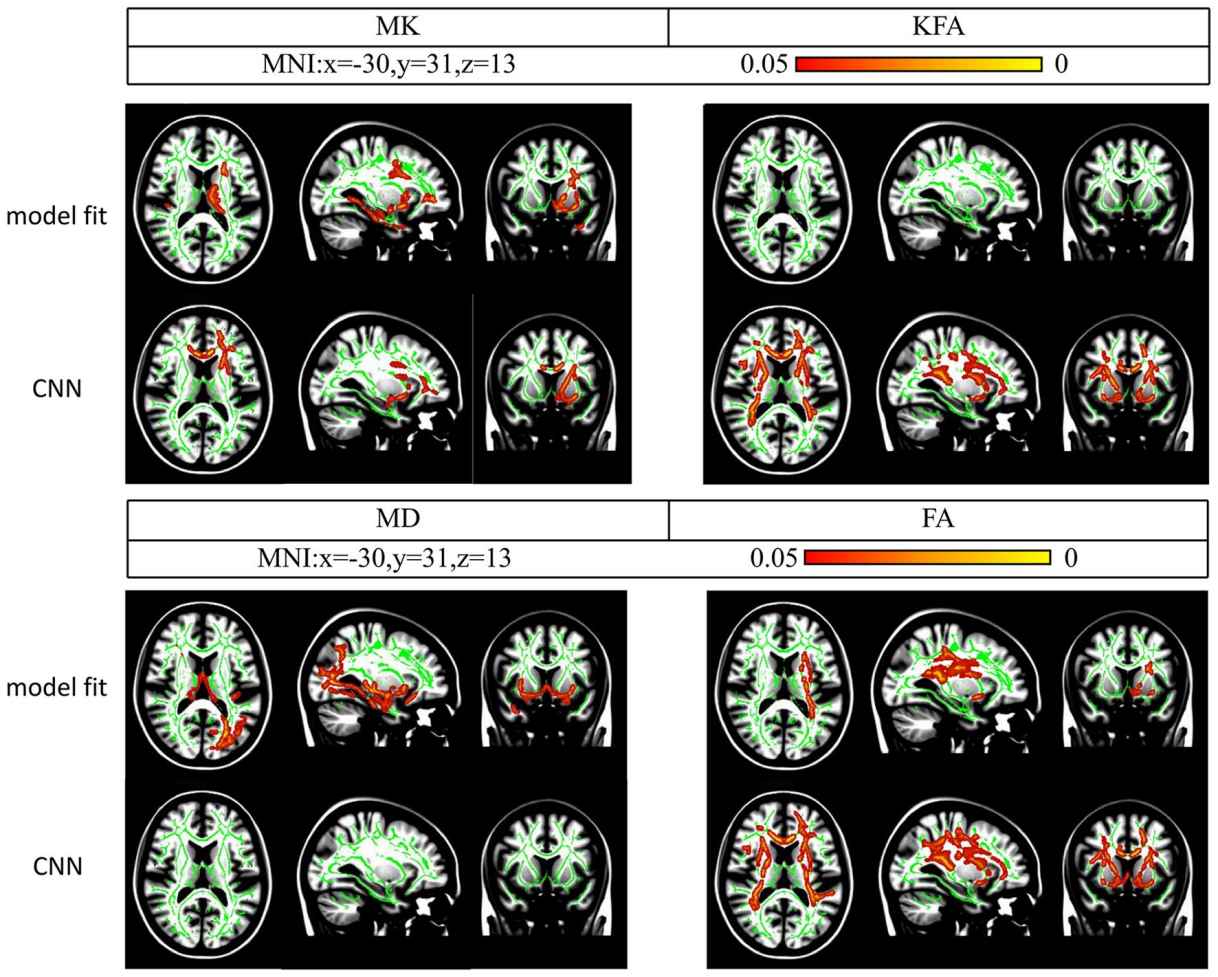
Tract-based spatial statistics analysis of diffusion kurtosis imaging measures using the CNN-based and model-fitting methods. Note. MK = mean kurtosis

Please check Fig. 5 and Additional file 2: Table S2 for more details.

#### CNN-based method

MK values in the forceps minor, left IFOF, left UNC and left ATR, et al. were significantly increased in PD patients compared to HCs.

Further, PD patients showed higher KFA and FA values in multiple brain regions, such as the bilateral ATR, bilateral IFOF, bilateral SLF, and forceps minor et al. than HCs.

We did not find a significant difference in MD values between the two groups (*p* < 0.05, FWE-corrected).

Please check Fig. 5 and Additional file 2: Table S2 for more details.

### Correlation analysis

#### Model-fitting method

We did not find any significant correlation between the DKI scalar measures and clinical assessments in patients with PD.

#### CNN-based method

**Fig. 6:**
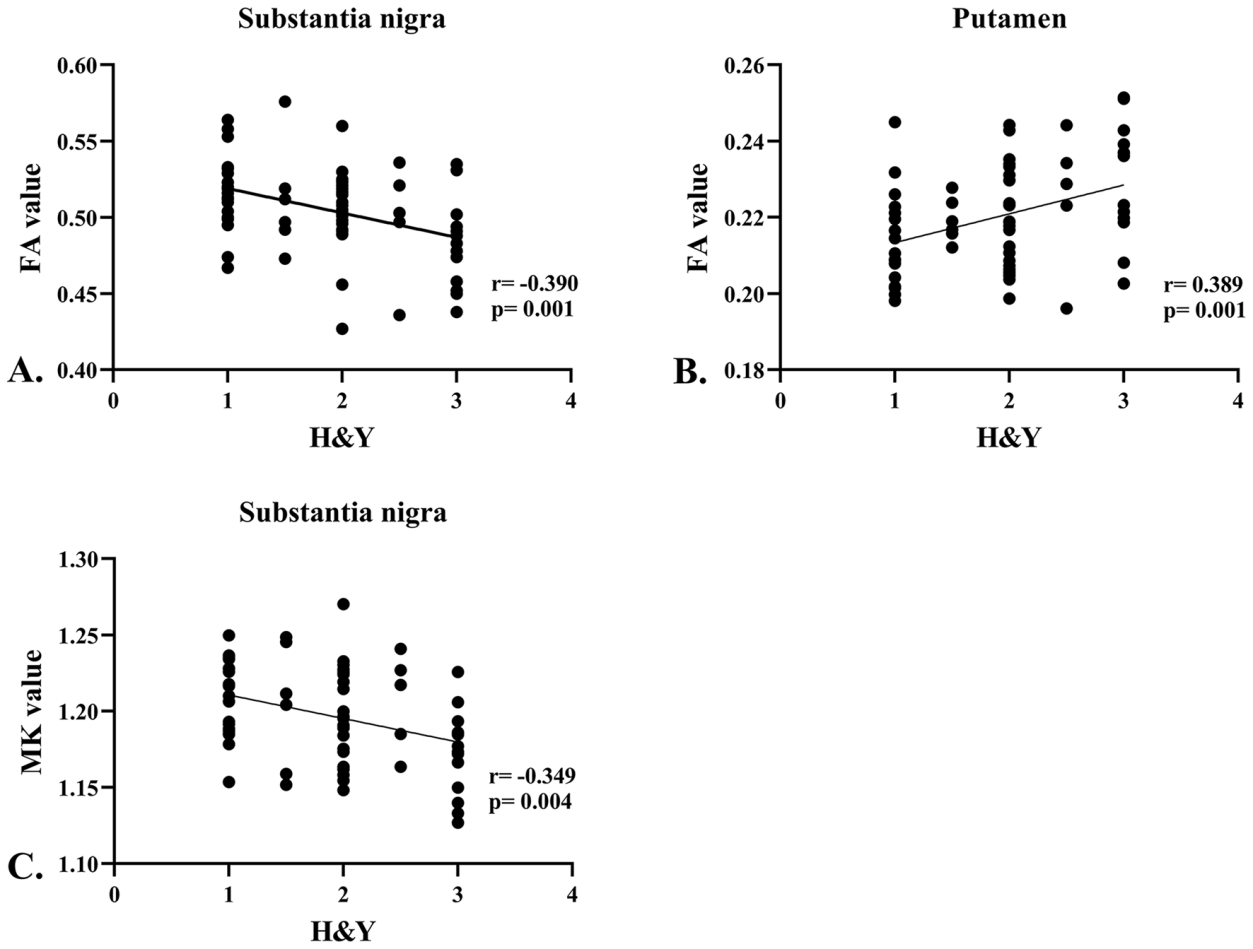
Spearman's correlation between the diffusion kurtosis imaging scalar measures and Hoehn and Yahr scales. **A**. Negative correlation between the fractional anisotropy (FA) values in the substantia nigra and Hoehn and Yahr (H&Y) scales. **B**. Positive correlation between the FA values in the putamen and H&Y scales. **C**. Negative correlation between the mean kurtosis values in the substantia nigra and H&Y scales

We found a positive correlation between the FA values of the left putamen and H&Y scales (r = 0.389, *p* = 0.001). A negative correlation was observed between the H&Y scales and FA and MK values in the right SN (r = − 0.390, *p* = 0.001; and r = − 0.349, *p* = 0.004, respectively) (Fig. 6).

## Discussion

The major finding of this study was that the CNN-estimated MD values in the left SN and bilateral Cau were increased in PD patients compared to HCs. Additionally, the CNN-estimated FA and MK values in the right SN were negatively correlated with the H&Y scales, and CNN-estimated FA values in the left Put were positively correlated with the H&Y scales. In contrast, with the model-fitting method, there was no significant difference in MD values in the SN between PD patients and HCs, and there was no significant correlation between DKI scalar measures and clinical assessments in PD patients. Our findings suggest that the CNN-based method has the potential to optimize the estimation of DKI scalar measures and improve the sensitivity to detect PD-related imaging features.

In this study, we trained the CNN with data from five healthy volunteers and tested the trained network on another two healthy subjects. The RMSE and PSNR results suggested that the CNN-based method provided more accurate DKI scalar measures than the conventional model-fitting method. However, we failed to obtain ground truth data on PD patients due to limitations in clinical settings and the inconvenience for PD patients to travel. The rationale for applying a trained network to patient data is that the relationships between the original DW-images and the corresponding DKI scalar measures were learned based on voxel-wise diffusion data. That is, the CNN provided estimations of DKI in every voxel and was sensitive to different diffusion signals. Therefore, it is natural to assume that the trained CNN was capable of estimating DKI scalar measures from another diffusion dataset that shared common acquisition parameters with the training dataset.

Using the CNN-based method, we found greater MD values in several brain regions in PD patients than in HCs, particularly in the left SN, which is consistent with previous reports using region of interest (ROI) analysis [10, 11, 14, 33–35]. PD is characterized by the progressive death of dopaminergic neurons in the SN, followed by the loss of dopaminergic projections from the SN to the striatum, resulting in a series of motor and non-motor symptoms [36]. According to the mathematical concept of a tensor, the three-dimensional shape of the diffusion elliptical structure depends on three eigenvalues (λ1, λ2, λ3) of orthogonal principal axes without directions. The MD value was the average of the three eigenvalues. The impaired axons and neurons and loss of myelin integrity in PD patients result in a decrease in the restriction of water molecule displacement, which induces increased MD values [35, 37]. Regionally increased MD values in the left SN and bilateral Cau estimated by the CNN method were consistent with the pathological lesions in PD patients. In contrast, we did not find increased MD values in the SN in patients with PD compared to HCs by applying the model-fitting method. This finding indicates that the CNN-based method can better reveal the pathological features of PD than the model-fitting method.

We did not observe modulation of FA and MK values in the SN in PD patients, which is in line with a previous report [14]. In contrast, some previous studies based on ROI analysis showed decreased or increased FA and/or increased MK in the SN in PD patients [6, 34]. We speculate that different analysis methods may be responsible for these controversial results. The whole-brain unpaired t-test, moving beyond the hypothesis-driven ROI analysis, focused the statistical information on each voxel accompanied with increased partial volume effects and false-positive risk, particularly within the pathological brain tissues. Moreover, we suggest that these controversial findings may be due to the heterogeneity of recruited patients and variations in imaging quality [4, 8]. In addition, it has been reported that iron deposition could increase FA values and decrease MD values in white and gray matter [38]. Numerous reports have demonstrated iron accumulation in the SN [39–41]. Thus, different levels of iron deposition in the SN may also have contributed to these inconsistent findings.

We found a negative correlation between the H&Y scales and CNN-estimated FA values and MK values in the SN, as well as a positive correlation between the H&Y scales and CNN-estimated FA values in the Put. These results indicated that FA and MK in the SN decreased, while FA in the Put increased with disease progression. As most of our patients were in the early stages (55 of our patients were at H&Y stages 1 and 2), it is possible to detect decreased FA in the SN if more advanced patients were enrolled. We did not find any significant correlation between DKI scalar measures and clinical assessments in PD patients using the model-fitting method, which further proves that using the CNN-based method to estimate DKI measures can improve the ability to explore PD-related neural modulations compared to using the model-fitting method.

For the TBSS analysis, increased FA values were observed in the brain white matter, such as ATR, IFOF with both methods, which was in line with previous studies [13, 42–44]. It has been shown that increased FA in these white matter regions correlates with better olfactory function and lower motor severity [45]. Thus, the increased diffusional properties of white matter might reflect microstructural compensation [45].

We observed greater MK values in the white matter in PD patients, which is inconsistent with previous reports. Previous studies found no significant difference in MK values [46, 47], or decreased MK values in the anterior cingulum, IFOF, and UNC in PD patients. We suggest that the heterogeneity of recruited patients and differences in the protocol of DW-image acquisition and image processing may have contributed to these inconsistent findings. In addition, we found increased KFA in the white matter, which has not been reported previously. KFA values, resembling the FA definition, quantify the degree of anisotropy of non-Gaussian diffusion. In the current study, the increased KFA and FA values were observed in the same white matter fibers. To date, only a small number of studies have focused on kurtosis changes in the white matter in PD patients [7, 48], and it is necessary to perform large cohort studies to elucidate the microstructural changes in white matter in PD patients.

There are some limitations to this study, such as the use of healthy volunteer subjects, which only provided DKI estimations closer to the ground truth. While we have validated the assumption that the CNN trained network is applicable to the PD datasets, further efforts will be made to include more ground truth data and more subjects to obtain more accurate results.

In conclusion, the CNN-based method has the potential to sensitively detect nigral pathology and improve the robustness and performance of DKI with few DW-images, and then to differentiate PD patents from HCs. In addition, compared with the model-fitting method, the CNN-based method can better determine the relationship between DKI parameter measures and clinical assessment susceptibility. These findings confirm that the CNN can contribute to the determination of PD-associated imaging features.

## Supplementary Information

**Additional file 1:Table S1.** Summary of the t-test results of the comparison between the two groups.

**Additional file 2:Table S2.** Summary of the TBSS results of the comparison between the two groups.

## Data Availability

The datasets used and/or analyzed during the current study are available from the corresponding author on reasonable request.

## Abbreviations

Acc: Accumbens
ACR: Anterior corona radiata
AP: Anterior–posterior
ATR: Anterior thalamic radiation
Cau: Caudate
CNN: Convolutional neural network
DKI: Diffusion kurtosis imaging
DTI: Diffusion tensor imaging
DW-images: Diffusion-weighted images
DWI: Diffusion-weighted imaging
FA: Fractional anisotropy
FSL: FMRIB Software Library
FOV: Field of view
FWE: Family-wise error
GCC: Genu of corpus callosum
GP: Globus pallidus
H&Y: Hoehn and Yahr
HCs: Healthy controls
IFOF: Inferior fronto-occipital fasciculus
ILF: Inferior longitudinal fasciculus
KFA: Kurtosis fractional anisotropy
MD: Mean diffusivity
MDS-UPDRS III: Movement Disorder Society Unified Parkinson’s Disease Rating Scale, part III
MK: Mean kurtosis
MNI152: Montreal Neurological Institute 152
MRI: Magnetic resonance imaging
PA: Posterior–anterior
PD: Parkinson’s disease
PSNR: Peak signal-to-noise ratio
Put: Putamen
RF: Radiofrequency
RMSE: Root-mean-squared error
ROI: Region of interest
SCR: Superior corona radiata
SE-EPI: Spin-echo echo-planar imaging sequences
SLF: Superior longitudinal fasciculi
SMS-EPI: Simultaneous multi-slice diffusion echo-planar imaging sequence
SN: Substantia nigra
TBSS: Tract-based spatial statistics
TE: Echo time
TFCE: Threshold-free cluster enhancement
Thal: Thalamus
TR: Repetition time
