# Supplementary material for: Convolutional neural network optimizes the application of diffusion kurtosis imaging in Parkinson’s disease
Source: Brain Inform. 2021 Sep 28;8(1):18. doi: 10.1186/s40708-021-00139-z (PMC8479023; doi:10.1186/s40708-021-00139-z)
Supplement: Supplementary file 1 — Additional file 1:Table S1. Summary of the t-test results of the comparison between the two groups. [file 40708_2021_139_MOESM1_ESM.docx]

| Group Comparison  (PD˃HC) | DKI measures | Methods | Brain regions | | | |
| --- | --- | --- | --- | --- | --- | --- |
| **T-test** | MK | Model-fitting | na. | | | |
|  |  | CNN-based | na. | | | |
|  | KFA | Model-fitting | na. | | | |
|  |  | CNN-based | ACR_L | CC_L |  |  |
|  | FA | Model-fitting | Acc_L  ACR  ALIC  ATR  Cau_L | CC  CST_L  CWM  EC | FO  GP_L  IFOF  Put | SCR  SFOF_R  SLF  UNC_L |
|  |  | CNN-based | ACR  ALIC_L  ATR  Cau_L  CC | CWM  CST_L  EC_L  IFOF  PCR_L | Put_L  SCR  SFOF_L  SLF  UNC_L |  |
|  | MD | Model-fitting | Acc  ACR  ALIC_R  Amyg  ATR  BCC  Cau  CC | CGC  CGH  CP_L  CST  CWM  EC  FO  FX | GCC  GP  Hippo  ICP  IFOF  ILF  MCP  PCR | PLIC  RLIC  SCR  SLF  TAP  Thal  UNC  PTR |
|  |  | CNN-based | Acc  ALIC_L  Amyg  ATR  BCC  Cau  CC  CGC | CGH  CST  CWM  FO  FX  GP  Hipp | ICP_R  IFOF  MCP  PCR_L  PLIC_L  PTR  Put | SCR  SFOF  SLF  SN_L  TAP  Thal  UNC |

**Supplementary Table 1.** Summary of the t-test results of the comparison between the two groups.

*Note.* The indicated side represents bilateral brain regions.

L=left; R=right; Acc=Accumbens; ALIC=Anterior limb of internal capsule; Amyg=amygdala; ATR=Anterior thalamic radiation; BCC=Body of corpus callosum; Caud=Caudate; CC=Cerebral Cortex; CGC=Cingulum Cingulate Gyrus; CGH=Cingulum Hippocampal; CST=Corticospinal tract; CWM=Cerebral White Matter; EC=External capsule; FO=forceps; FX=Fornix (cres); GCC=Genu of corpus callosum; GP=Globus Pallidum; Hippo=hippocampus; ICP=Inferior cerebellar peduncle; IFOF=Inferior fronto-occipital fasciculus; ILF=inferior longitudinal fasciculus; MCP=Middle cerebellar peduncle; PCR=Posterior corona radiata; PLIC=Posterior limb of internal capsule; PTR=Posterior thalamic radiation; Put=putamen; RLIC=Retrolenticular part of internal capsule; SCP=Superior cerebellar peduncle; SCR=Superior corona radiata; SFOF=Superior fronto-occipital fasciculus; SLF=Superior longitudinal fasciculus; SN=Substantia nigra; TAP=Tapatum; Thal=thalamus; UNC=Uncinate fasciculus.

na.=not applicable
