# Supplementary material for: Convolutional neural network optimizes the application of diffusion kurtosis imaging in Parkinson’s disease
Source: Brain Inform. 2021 Sep 28;8(1):18. doi: 10.1186/s40708-021-00139-z (PMC8479023; doi:10.1186/s40708-021-00139-z)
Supplement: Supplementary file 2 — Additional file 2:Table S2. Summary of the TBSS results of the comparison between the two groups. [file 40708_2021_139_MOESM2_ESM.docx]

| Group Comparison  (PD˃HC) | DKI measures | Methods | Brain regions | | |
| --- | --- | --- | --- | --- | --- |
| **TBSS** | MK | Model-fitting | ATR_L  CST_L  CGC_L | CGH_L  FO  IFOF_L | ILF  SLF_L  UNC_L |
|  |  | CNN-based | ATR_L  CGC_L  FO | IFOF_L  ILF_L | SLF_L  UNC_L |
|  | KFA | Model-fitting | na. | | |
|  |  | CNN-based | ATR  CST  CGC | CGH  FO  IFOF | ILF  SLF  UNC |
|  | FA | Model-fitting | ATR  CST_L  CGC_L | FO  IFOF_L  ILF_L | SLF_L  UNC_L |
|  |  | CNN-based | ATR  CST  CGC | CGH  FO  IFOF | ILF  SLF  UNC |
|  | MD | Model-fitting | ATR  CST  CGC | CGH  FO  IFOF | ILF  SLF  UNC |
|  |  | CNN-based | na. | | |

**Supplementray Table 2.** Summary of the TBSS results of the comparison between the two groups.

Note. The indicated side represents bilateral brain regions.

L=left; R=right; ATR=Anterior thalamic radiation; CGC=Cingulum Cingulate Gyrus; CGH=Cingulum Hippocampal; CST=Corticospinal tract; FO=forceps; IFOF=Inferior fronto-occipital fasciculus; ILF=inferior longitudinal fasciculus; SLF=Superior longitudinal fasciculus; UNC=Uncinate fasciculus.

na.=not applicable
